# Hypocretin receptor 1 blockade early in abstinence prevents incubation of cocaine seeking and normalizes dopamine transmission

**DOI:** 10.1038/s41386-025-02315-9

**Published:** 2026-02-04

**Authors:** Philip J. Clark, Volodar M. Migovich, Sanjay Das, Wei Xu, Yanan Zhang, Sandhya Kortagere, Rodrigo A. España

**Affiliations:** 1 Department of Neurobiology and Anatomy, Drexel University College of Medicine, Philadelphia, PA 19129 USA; 2 Department of Microbiology and Immunology, Drexel University College of Medicine, Philadelphia, PA 19129 USA; 3 Research Triangle Institute, Research Triangle Park, P.O. Box 12255, Durham, NC 27709 USA

**Keywords:** Motivation, Cellular neuroscience

## Abstract

Abstinence from cocaine use has been shown to elicit a progressive intensification or incubation of cocaine craving/seeking that is posited to increase the likelihood of relapse. While the mechanisms underlying incubation of cocaine seeking remain elusive, considerable evidence suggests that abstinence from cocaine promotes mesolimbic dopamine adaptations that contribute to exaggerated cocaine seeking. Consequently, preventing these dopamine adaptations may reduce incubation of cocaine seeking and thereby decrease the likelihood of relapse. In the present studies, we first examined the relationship between incubation of cocaine seeking and dopamine transmission in the nucleus accumbens following abstinence from intermittent access to cocaine. Given the extensive evidence that hypocretins/orexins regulate motivation for cocaine, we then examined to what extent an intraperitoneal injection of a hypocretin receptor 1 antagonist on the first day of abstinence would prevent incubation of cocaine seeking and dopamine adaptations later in abstinence. Results indicated that abstinence from intermittent access to cocaine engendered robust incubation of cocaine seeking in both female and male rats. We also observed aberrant dopamine transmission, but only in rats that displayed incubation of cocaine seeking. Further, we showed that a single injection of the hypocretin receptor 1 antagonist, RTIOX-276, on the first day of abstinence prevented incubation of cocaine seeking and aberrant dopamine transmission. These findings suggest that hypocretin receptor 1 antagonism may serve as a viable therapeutic for reducing cocaine craving/seeking early in abstinence, thus reducing the likelihood of relapse.

## Introduction

In humans, abstinence from drug use is associated with a progressive intensification—or incubation—of drug craving/seeking [1–3] that is hypothesized to promote relapse to drug use [4]. This intensification of drug craving has been modeled preclinically as increased cue-induced drug seeking across abstinence, leading to the hypothesis that abstinence induces neuroadaptations that underlie the incubation of drug seeking [5–11].

Accumulating evidence implicates the mesolimbic dopamine pathway in the development of incubation of cocaine seeking [12–16]. For example, using the intermittent access (IntA) schedule of reinforcement—which produces a pattern of cocaine exposure that more closely reflects the intermittent nature of human use—we observed robust incubation of cocaine seeking and enhanced dopamine transporter (DAT) function in the nucleus accumbens (NAc) core after 28 days of abstinence [17]. These and related observations suggest that incubation of cocaine seeking may be linked to DAT adaptations and that normalizing dopamine transmission during abstinence may be an effective strategy for reducing cocaine seeking. Unfortunately, pharmacotherapies that target dopamine systems directly are largely ineffective and may have abuse potential themselves [18].

Extensive evidence indicates that the hypocretin/orexin peptides participate in reward and reinforcement and regulate dopamine transmission. For example, hypocretin-1 peptide increases motivation for cocaine [19] and reinstates cocaine-seeking [20, 21], while hypocretin receptor 1 (Hcrtr1) antagonism decreases motivation for cocaine [22–28] and blocks reinstatement of cocaine-seeking [29–31]. Moreover, knockout of hypocretin peptides reduces cocaine conditioned place preference [32], and knockdown of Hcrtr1 reduces motivation for cocaine [33, 34].

The behavioral effects of hypocretin manipulations involve alterations in mesolimbic dopamine transmission. Hypocretin neurons innervate the dopamine-rich ventral tegmental area (VTA), where the hypocretin-1 peptide potentiates excitatory drive and increases dopamine neuron firing [22, 25, 35–41]. Further, acute administration of Hcrtr1 antagonists decreases dopamine in the NAc under baseline conditions and reduces dopamine responses to cocaine [23, 24, 28, 42]. Although these observations have been instrumental in establishing the involvement of hypocretin in motivation for cocaine, to date there have been no reports examining the effects of Hcrtr1 disruption during periods of abstinence on incubation of drug seeking.

Here, we first examined to what extent IntA to cocaine promoted incubation of cocaine seeking and dopamine adaptations in the NAc after a relatively short, 8-day abstinence period. This early timepoint was selected to determine whether incubation emerges rapidly following IntA to cocaine. Female and male Long Evans rats self-administered cocaine on an IntA schedule and then underwent 8 days of abstinence. On abstinence day 1 (AD1) and abstinence day 8 (AD8), rats were tested on cue-induced cocaine seeking tests to assess incubation of cocaine seeking. The following day, we used fast-scan cyclic voltammetry (FSCV) to examine alterations in dopamine transmission and Western blotting to measure DAT expression and phosphorylation.

We then explored the utility of a single injection of the Hcrtr1 antagonist—RTIOX-276—administered on AD1 for preventing incubation of cocaine seeking and associated dopamine adaptations. We chose this early treatment timepoint based on our prior work indicating that a single injection of RTIOX-276 on the first day of abstinence reduced demand for cocaine after 7 days of abstinence from IntA to cocaine [43]. Rats self-administered cocaine on the IntA schedule and were tested for cue-induced seeking on AD1, immediately followed by intraperitoneal treatment with vehicle or 20 mg/kg RTIOX-276. Rats were then left undisturbed until AD8, when they were retested for cue-induced seeking. The following day, FSCV and Western blotting were used to assess dopamine transmission and DAT expression in the NAc. We hypothesized that RTIOX-276 treatment on AD1 would attenuate incubation of cocaine seeking and dopamine adaptations early in abstinence from IntA to cocaine.

## Materials and methods

### Animals

Adult female (220–260 g) and male (300–410 g) Long Evans rats (Envigo, Frederick, MD, USA) were maintained on a 12 h reverse light/dark cycle (1500 lights on; 0300 lights off) with ad libitum access to food and water. After arrival, rats were given at least 7 days to acclimate to the animal facility prior to surgery. All protocols and animal procedures were conducted in accordance with the NIH Guide for the Care and Use of Laboratory Animals under supervision of the IACUC at Drexel University.

### Drugs

Cocaine hydrochloride was provided by the National Institute on Drug Abuse Drug Supply Program (Research Triangle Park, NC, USA). For self-administration experiments, cocaine was dissolved in 0.9% saline. For FSCV experiments, cocaine was dissolved in artificial cerebrospinal fluid (aCSF). RTIOX-276 is a highly selective Hcrtr1 antagonist with a Ki of approximately 8.5 nM for Hcrtr1 and negligible affinity (>10,000 nM) for hypocretin receptor 2 [44, 45]. The metabolic stability of RTIOX-276 in rat liver microsomes suggests a short half-life of less than 10 min (unpublished observations). RTIOX-276 (20 mg/kg i.p.) was dissolved in a 1.7 mL solution of 5% Tween20 in H20 + 1 M HCl before the addition of 0.3 ml of 1 M NaOH with a final pH of ~7.6 [23, 44].

### Intravenous catheter surgery

Rats were anesthetized using 2.5% isoflurane and implanted with a silastic catheter (Access Technologies, Skokie, IL) into the right jugular vein for intravenous delivery of cocaine. The catheter was connected to a cannula which exited through the skin on the dorsal surface in the region of the scapulae. Ketoprofen (Patterson Veterinary, Devens, MA; 5 mg/kg s.c.) and Enrofloxacin (Norbrook, Northern Ireland; 5 mg/kg s.c.) were provided at the time of surgery and a second dose was given 12 h later. Antibiotic/analgesic powder (Zoetis, Kalamazoo, MI) was applied around the chest and back incisions. Rats were subsequently singly housed and allowed to recover for 5 days prior to self-administration. Intravenous catheters were manually flushed with gentamicin (5 mg/kg i.v.; Vedco, St. Joseph, MO) in heparinized saline every day during recovery.

### Self-administration

Self-administration chambers contained two levers – one designated active and the other as inactive. All measures were recorded using custom created Ghost Software [46]. Rats lived in the self-administration chambers 7 days a week and were not handled throughout experimentation. Each rat underwent one cocaine self-administration session per day from 10:00–16:00.

### Acquisition

Rats trained to self-administer cocaine using a long access schedule wherein rats had cocaine access for 6 h/day under a fixed ratio 1 (FR1) schedule. During this training, single active lever responses initiated an i.v. injection of cocaine (0.75 mg/kg, over 2.5 s–4.5 s) paired with a cue light above the active lever and a 20-sec timeout during which levers retracted [17, 47, 48]. Responses on the inactive lever were recorded but otherwise had no consequence. Rats were considered to have acquired self-administration after reaching ≥ 40 infusions in one session but were not switched to IntA self-administration until they maintained ≥ 40 infusions for two sessions. Rats were removed from the study if they did not meet these criteria within 10 days of beginning self-administration.

### Intermittent access

Following acquisition, rats were switched to the IntA schedule of reinforcement [48]. We selected the IntA procedure over other schedules of reinforcement because it more closely models the temporal patterns of human cocaine use, produces robust cocaine-seeking behavior, and induces dopamine transmission changes that may be unique to this procedure [16, 17, 47–49]. During IntA sessions, rats had access to cocaine for 5 min followed by a 25-min timeout during which both levers retracted. These 30-min trials repeated for 12 trials/session for a total of 6 h/day. Active lever responses resulted in a single i.v. injection of cocaine (0.375 mg/kg over 0.7–1.2 s), paired with a cue light above the active lever. Inactive lever responses were recorded but had no consequence. Unlike training sessions, there was no timeout following active lever presses during the 5-min access periods [17]. Rats underwent IntA for 7 consecutive days before moving to abstinence.

#### Abstinence and cue-induced seeking tests

Following the final IntA session, rats underwent an 8-day forced abstinence period. To assess cocaine seeking, rats performed cue-induced drug seeking tests on AD1 and AD8. Cue-induced seeking tests were 1 h in length and all cues (lights, lever presentation, etc.) were identical to the training sessions, except that active lever responses did not result in a cocaine infusion. We used this within-subjects design to assess individual differences in cocaine seeking changes over the course of abstinence, similar to what has been reported previously [17, 50–56]. In a subset of analyses, we separated rats into two groups; one group that displayed greater pressing on AD8 than on AD1 (incubated) and another group that did not increase pressing on AD8 relative to AD1 (non-incubated). For the RTIOX-276 studies, rats were treated with intraperitoneal vehicle or 20 mg/kg RTIOX-276 immediately following the AD1 seeking test.

### Ex vivo fast-scan cyclic voltammetry

18 h after the AD8 seeking test, rats were prepared for FSCV experiments. Cocaine-naive rats did not receive catheterization surgeries and were used as experimental controls. Similar to rats that self-administered cocaine, naive control rats were not handled daily and were housed in the same room. On the day of FSCV experiments, rats were anesthetized with 2.5% isoflurane for 5 min and brains were rapidly dissected and transferred to ice-cold aCSF containing NaCl (126 mM), KCl (2.5 mM), NaH2PO4 (1.2 mM), CaCl2 (2.4 mM), MgCl2 (1.2 mM), NaHCO3 (25 mM), glucose (11 mM), and L-ascorbic acid (0.4 mM), with pH adjusted to 7.4. A vibratome was used to produce 400 µm-thick coronal sections containing the NAc. Slices were transferred to room temperature oxygenated aCSF and left to equilibrate for at least 1 h before being transferred into a recording chamber with aCSF (32°C).

A bipolar stimulating electrode was placed on the surface of the tissue in the NAc, and a carbon fiber microelectrode was implanted between the stimulating electrode leads. Dopamine release was evoked every 3 min using a single electrical pulse (400 µA, 4 ms, monophasic) and measured using Demon Voltammetry and Analysis Software [57]. Once baseline dopamine release was stable (3 successive stimulations within <10% variation), the slice was exposed to increasing cocaine concentrations (0.3, 1, 3, 10, 30 µM). FSCV data were analyzed using Michaelis-Menten kinetic methods to calculate the maximal rate of dopamine uptake (Vmax) and DAT sensitivity to cocaine (i.e., cocaine-induced inhibition of dopamine uptake; apparent Km) [57].

### Western blotting

Synaptosomes were prepared, and membrane fractionation was performed using a modification of published procedures [42, 58, 59]. The ventral striatum was dissected and stored at -80°C until preparation. Tissue was homogenized in ice-cold lysis buffer (1000 ml, 50 mM Tris-HCl, pH 7.4, 1 mM EDTA, 320 mM sucrose) with 1x protease inhibitor cocktail, 1x phosphatase inhibitor cocktail, and 1 mM PMSF. The homogenate was centrifuged at 1000x g for 5 min at 4°C. The supernatant was re-centrifuged at 10,000x g for 20 min at 4°C. The resulting synaptosomal pellet was resuspended with 300 ml lysis buffer. Immunoblotting was performed with rabbit anti DAT polyclonal antibody (1:1000, EMD Millipore), rabbit anti phospho-DAT polyclonal antibody (1:1000; P435-53, PhosphoSolutions), peroxidase-conjugated goat anti rabbit IgG (H1 L) (1:5000; 111-035-144, Jackson ImmunoResearch Laboratories), and anti GAPDH polyclonal antibody (1:5000; PA1-987, Thermo Fisher Scientific). Total DAT (tDAT), phosphorylated DAT at threonine 53 (pDAT), and GAPDH immunoblots were quantified by densitometry with ImageQuant LAS4000 (GE Healthcare Bio-Sciences). GAPDH was used as a housekeeping protein to control for variability in sample loading and hence pDAT and tDAT levels were normalized to GAPDH levels for quantification [42, 58, 59]. Additionally, we presented the proportion of total DAT that are phosphorylated at the threonine 53 site by quantifying pDAT/tDAT.

### Statistical analysis

Analyses to detect sex differences were conducted for all behavioral, FSCV, and Western blotting metrics evaluated here (see Supplemental Table I and Table II). We did not observe any interactions between sex and measures of interest, indicating that both females and males responded similarly across test conditions. Therefore, female and male data were combined as recommended by prior studies [60–62] and our own work [17, 34].

Behavioral data were analyzed using paired or unpaired t-tests or two-way ANOVAs. Baseline FSCV measurements of dopamine peak height and uptake and Western blotting data were analyzed using unpaired t-tests or one-way ANOVAs. FSCV measurement of dopamine responses to cocaine were analyzed using two-way ANOVAs. When significant effects were detected, Holm-Bonferroni post-hoc tests were performed. All analyses were conducted using GraphPad Prism 10.

## Results

### Intermittent access to cocaine promoted incubation of cocaine seeking in a subset of rats

**Fig. 1:**
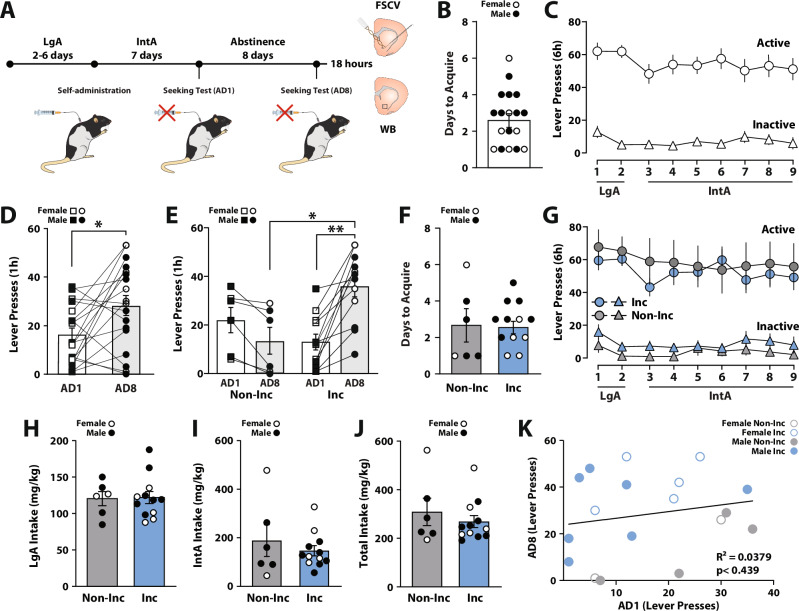
Abstinence from IntA to cocaine promoted incubation of cocaine seeking. **A** Experimental timeline for behavioral, fast-scan cyclic voltammetry, and Western Blot data. **B** Number of days to acquire cocaine self-administration for all rats. **C** Active and inactive lever presses across self-administration sessions for all rats. **D** Active lever presses during cue-induced seeking tests for all rats. **E** Active lever presses during cue-induced seeking tests for non-incubated (Non-Inc) and incubated (Inc) rats. **F** Number of days to acquire cocaine self-administration for Non-Inc and Inc rats. **G** Active and inactive lever presses across self-administration sessions for Non-Inc and Inc rats. Cocaine intake during **H** long access (LgA), **I** intermittent access (IntA), and **J** combined LgA and IntA self-administration. **K** Pearson correlation between AD1 and AD8 active lever pressing. Data shown as mean ± SEM. ○ females, ● males. Student’s t-test, *p < 0.05. Holm-Bonferroni, *p < 0.05, **p < 0.01.

To examine to what extent IntA to cocaine elicits incubation of cocaine seeking, rats self-administered on the IntA schedule for 7 consecutive days, followed by cue-induced drug seeking tests on AD1 and AD8 (Fig. 1A). Rats acquired self-administration (1 day of >40 presses) in 2.72 ± 0.35 days (Fig. 1B) and displayed robust cocaine intake during the last 2 days of long access training and the 7 days of IntA self-administration (Fig. 1C). A paired Student’s t-test between the last day of IntA and the first day of IntA revealed that IntA did not produce escalation of cocaine intake (*t*(17) = 0.6796, *p* < 0.5059). By comparison, a paired Student’s t-test comparing lever pressing on AD1 to AD8 showed that as a group, rats pressed significantly more on AD8 (*t*(17) = 2.728, *p* < 0.0143), indicating robust incubation of seeking (Fig. 1D). Closer examination of the data revealed that not all rats displayed greater pressing on AD8 than on AD1, which indicated that only a subset of rats displayed incubation of cocaine seeking. Therefore, we separated rats into two groups; one group that increased lever pressing on AD8 relative to AD1 (incubated rats), and a second group that did not increase lever pressing on AD8 (non-incubated rats). As expected based on this separation, a two-way ANOVA with incubation group (non-incubated vs. incubated) as the between-subjects variable and abstinence day (AD1 vs. AD8) as the within-subjects variable revealed a significant effect of day (*F*(1,16) = 5.531, *p* < 0.0318), a significant day x incubation group interaction (*F*(1,16) = 26.60, *p* < 0.001), but no effect of incubation group (*F*(1,16) = 1.386, *p* < 0.2563) on lever pressing during cue-induced cocaine seeking tests. Holm-Bonferroni post-hoc tests showed that incubated rats displayed greater pressing on AD8 compared to AD1 (*p* < 0.0001) and also compared to rats that did not incubate on AD8 (*p* < 0.0015; Fig. 1E). Notably, incubated rats did not differ from non-incubated rats on AD1 pressing (*p* < 0.1411) and while non-incubated rats did not significantly reduce lever pressing between AD1 and AD8, there was a strong trend for significance (*p* < 0.0504).

To assess whether the presence of incubation on AD8 was a consequence of potential differences in cocaine self-administration behavior prior to abstinence, we compared the number of days to acquire self-administration and cocaine intake between incubated and non-incubated subgroups. A Student’s t-test showed that there were no differences in the number of days to acquire between non-incubated vs. incubated groups (*t*(16) = 0.1087, *p* < 0.9148; Fig. 1F). Further, a two-way ANOVA with incubation group (non-incubated vs. incubated) as the between-subjects variable and day of self-administration (days 1-9) as the within-subjects variable revealed no significant effect of day (*F*(3.521,56.34) = 1.131, *p* < 0.3486) or incubation group (*F*(1,16) = 0.3440, *p* < 0.5657) and no day x incubation group interaction (*F*(3.521,56.34) = 0.4382, *p* < 0.7569) on lever pressing during the last 2 days of long access and the 7 days of IntA self-administration (Fig. 1G).

Similarly, there were no differences in the amount of cocaine intake for either the long access training (*t*(16) = 0.1137, *p* < 0.9109; Fig. 1H), the IntA self-administration (*t*(16) = 0.7642, *p* < 0.4559; Fig. 1I), or the combined intake during these two phases of self-administration (*t*(16) = 0.7619, *p* < 0.4572; Fig. 1J). Finally, we examined if the number of lever presses on AD1 predicted lever pressing on AD8 using a Pearson correlation and found no significant relationship between AD1 and AD8 lever pressing (*F*(1,16)  =  0.6300; R^2^  =  0.0379, *p* < 0.4390; Fig. 1K). Together, these results suggest that acquisition, intake, and AD1 seeking behavior did not differ between incubated and non-incubated rats.

#### Incubation of cocaine seeking enhanced dopamine transporter function

**Fig. 2:**
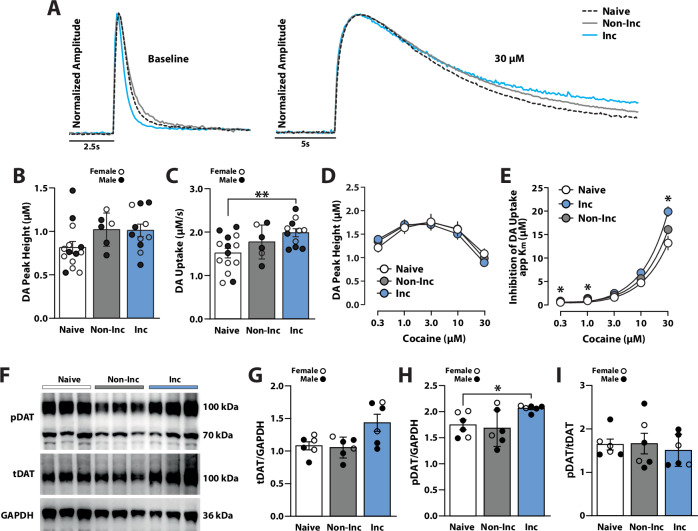
Abstinence from IntA to cocaine enhanced dopamine uptake, DAT sensitivity to cocaine, and pDAT expression in the nucleus accumbens. **A** Example dopamine traces for baseline measurements and following 30 μM cocaine. **B** Baseline dopamine peak height and **C** dopamine uptake for naive, non-incubated (Non-Inc), and incubated (Inc) rats. **D** Cocaine-induced dopamine peak height and **E** inhibition of dopamine uptake for naive, Non-Inc, and Inc rats. **F** Example Western blots. **G** Quantification of total membrane DAT (tDAT) over GAPDH, **H** phosphorylated DAT (pDAT) over GAPDH, and **I** pDAT over tDAT (pDAT/tDAT) for naive, Non-Inc, and Inc rats. Data shown as mean ± SEM. ○ females, ● males. Holm-Bonferroni, *p < 0.05, **p < 0.01 Inc vs. Naive.

To assess if incubation of cocaine seeking was associated with changes in dopamine transmission, we investigated evoked dopamine release (dopamine peak height) and uptake dynamics 18 h following the AD8 seeking tests (Fig. 1A). Cocaine naive rats were used as controls. A one-way ANOVA with incubation group (naive, non-incubated vs. incubated) as the between-subjects variable revealed no significant effect of incubation group (*F*(2,28) = 2.651, *p* < 0.0882) on dopamine peak height (Fig. 2B), but there was a significant effect of incubation group on dopamine uptake (*F*(2,28) = 4.792, *p* < 0.0162). Holm-Bonferroni post-hoc analyses indicated that incubated rats showed significantly higher dopamine uptake than naive rats (*p* < 0.0098) with no significant difference (*p* < 0.2228) between non-incubated and naive rats (Fig. 2C). These findings suggest that enhanced dopamine uptake during abstinence from IntA to cocaine is specific to rats that developed incubation of seeking.

To assess if incubation of cocaine seeking was associated with changes in dopamine responses to cocaine, we conducted FSCV with bath application of cocaine. A two-way ANOVA with incubation group (naive, non-incubated, vs. incubated) as the between-subject variable and cocaine concentration as the within-subject variable revealed a significant effect of concentration (Greenhouse-Geisser correction; *F*(2.151,58.09) = 91.05, *p* < 0.0001) but no significant effect of incubation group (*F*(2,27) = 0.0188, *p* < 0.9814) or an incubation group x concentration interaction (*F*(4.303,58.09) = 2.288, *p* < 0.0663), on cocaine-induced dopamine peak height, despite a strong trend (Fig. 2D). Finally, we examined whether DAT sensitivity to cocaine differed between incubated and non-incubated rats. A two-way ANOVA with incubation group (naive, non-incubated, vs. incubated) as the between-subjects variable and cocaine concentration as the within-subjects variable revealed a significant effect of concentration (*F*(1.124,30.34) = 332.3, *p* < 0.0001), incubation group (*F*(2,27) = 6.511, *p* < 0.0049), and a significant incubation group x concentration interaction (*F*(2.248,30.34) = 6.100, *p* < 0.0046) on DAT sensitivity to cocaine (Fig. 2E). Holm-Bonferroni tests showed that incubated rats displayed greater DAT sensitivity to cocaine compared to naive rats at the 0.3 µM (*p* < 0.0124), 1 µM (*p* < 0.0229), and 30 µM (*p* < 0.0227) concentrations (not significant at 3 µM, *p* < 0.0663 or 10 µM, *p* < 0.1090). By comparison there were no significant differences observed between non-incubated rats and naive controls for any concentration (0.3 µM, *p* < 0.7363; 1 µM, *p* < 0.3474, 3 µM, *p* < 0.8953; 10 µM, *p* < 0.3793; 30 µM, *p* < 0.4950). Finally, we examined if lever pressing on AD8 predicted changes in dopamine transmission using a Pearson correlation. Despite the finding that incubated rats displayed notable changes in dopamine transmission compared to naive controls, there were no significant relationships between lever pressing and dopamine peak height (*F*(1,15)  =  0.1036; R^2^  =  0.0068, p < 0.7519), dopamine uptake (*F*(1,15)  =  3.174; R^2^  =  0.1746, p < 0.0951), dopamine peak height following 30 µM cocaine (*F*(1,15)  =  0.7154; R^2^  =  0.0455, p < 0.4109), or inhibition of dopamine uptake following 30µM cocaine (*F*(1,15)  =  0.2667; R^2^  =  0.0175, *p* < 0.6131; Supplemental Fig. 1A–D).

#### Incubation of cocaine seeking promoted enhanced DAT expression and phosphorylation

We next asked if the observed enhancements in DAT function were associated with changes in DAT surface expression or phosphorylation. NAc tissue from naive controls and rats that underwent IntA to cocaine (Fig. 1A) were analyzed for total membrane DAT (tDAT) and DAT phosphorylated at threonine 53 (pDAT). A one-way ANOVA with incubation group (naive, non-incubated vs. incubated) as the between-subjects variable revealed a significant effect of incubation group on tDAT (*F*(2,15) = 5.36, *p* < 0.0175), pDAT (*F*(2,15) = 4.151, *p* < 0.0367) but not pDAT/tDAT (*F*(2,15) = 0.2254, *p* < 0.2802) expression (Fig. 2F–I). Holm-Bonferroni tests further demonstrated that incubated rats exhibited significantly greater pDAT levels than controls (*p* < 0.0181), a trend for an increase in tDAT (*p* < 0.0689), and no differences in pDAT/tDAT (*p* < 0.9999). Non-incubated rats did not differ from naive controls for either tDAT (*p* < 0.7775), pDAT (*p* < 0.7154), or pDAT/tDAT (*p* < 0.9369). We then examined if lever pressing on AD8 predicted changes in DAT biochemistry and we did not observe significant correlations for tDAT (*F*(1,11)  =  0.1398; R^2^  =  0.0125, *p* < 0.7156), pDAT (*F*(1,11)  =  1.410; R^2^  =  0.1136, *p* < 0.2601), or pDAT/tDAT (*F*(1,11)  =  2.260; R^2^  =  0.1705, *p* < 0.1609) expression (Supplemental Fig. 1E–G).

#### Hcrtr1 blockade early in abstinence prevented incubation of cocaine seeking

**Fig. 3:**
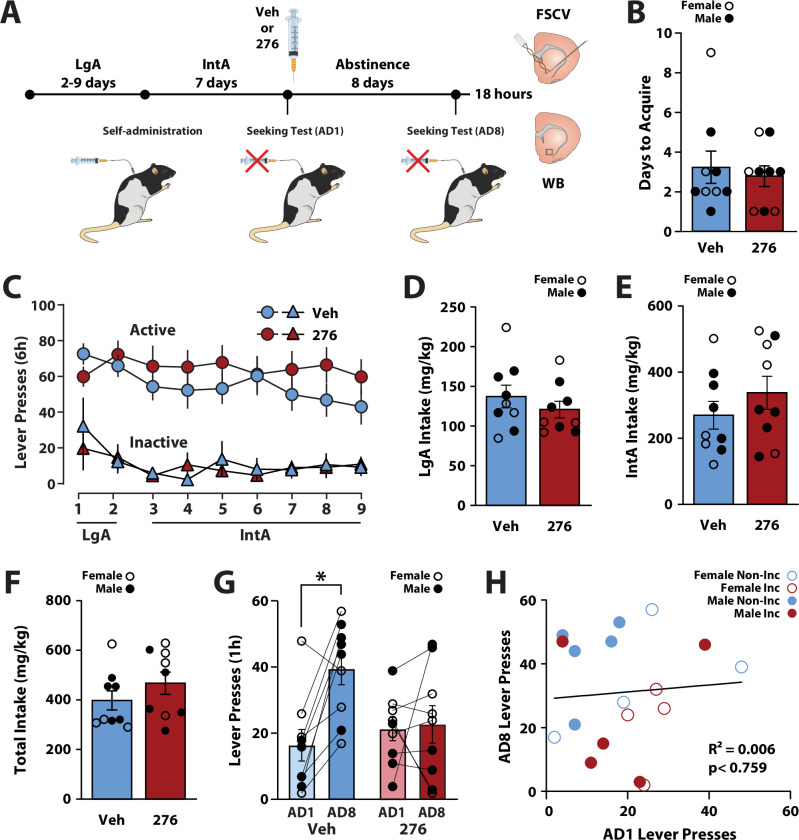
Hcrtr1 blockade prevented incubation of cocaine seeking. **A** Experimental timeline for behavioral, fast-scan cyclic voltammetry, and Western Blot data. **B** Number of days to acquire cocaine self-administration, **C** active and inactive lever presses during self-administration, cocaine intake during **D** long access (LgA), **E** intermittent access (IntA), and **F** combined LgA and IntA self-administration for rats that would later receive vehicle (Veh) or 20 mg/kg RTIOX-276 (276). **G** Active lever presses during cue-induced seeking tests and **H** Pearson correlation between AD1 and AD8 lever pressing for rats treated with vehicle and RTIOX-276. Data shown as mean ± SEM. ○ females, ● males. Holm-Bonferroni, *p < 0.05.

Extensive evidence indicates that disrupting Hcrtr1 signaling reduces behavioral and dopamine responses to cocaine [23, 24, 26, 28, 32–34]. Consequently, we investigated the effects of Hcrtr1 blockade on incubation of cocaine seeking and dopamine adaptations following abstinence from IntA to cocaine. Female and male rats underwent IntA self-administration and 8 days of abstinence (Fig. 3A). Immediately, after the seeking test on AD1, rats received a single i.p. injection of vehicle or 20 mg/kg of the Hcrtr1 antagonist RTIOX-276 [44]. A Student’s t-test revealed no differences in days to acquire cocaine self-administration between rats that would eventually receive vehicle or RTIOX-276 (t(16) = 0.4603, *p* < 0.6515; Fig. 3B). Similarly, a two-way ANOVA with future treatment (vehicle vs. RTIOX-276) as the between-subjects variable and day of self-administration (days 1–9) as the within-subjects variable revealed no effect of future treatment (*F*(1,16) = 0.7454, *p* < 0.4007), day (Greenhouse-Geisser Correction; *F*(3.762,60.19) = 1.759, *p* < 0.1526), or a future treatment x day interaction (*F*(3.762,60.19) = 1.660, *p* < 0.1743) on the number of injections taken during the last 2 days of long access training and the 7 days of IntA (Fig. 3C). With respect to escalation, a two-way ANOVA with future treatment as the between-subjects variable and day of IntA self-administration (days 3 vs. day 9) as the within-subjects variable revealed no effect of future treatment (*F*(1,16) = 1.139, *p* < 0.3016), day (*F*(1,16) = 3.479, *p* < 0.0806), or a future treatment x day interaction (*F*(1,16) = 0.3477, *p* < 0.5637) on cocaine intake (Fig. 3C). There were also no differences in the amount of cocaine intake for either the long access training (t(16) = 0.9269, *p* < 0.3678; Fig. 3D), the IntA self-administration (t(16) = 1.033, *p* < 0.3172; Fig. 3E), or the combined intake during these two phases of self-administration (t(16) = 1.179, *p* < 0.2555; Fig. 3F).

We then assessed whether Hcrtr1 blockade affected incubation of cocaine seeking. A two-way ANOVA with treatment (vehicle or RTIOX-276) as the between-subjects variable and seeking day (AD1 vs. AD8) as the within-subjects variable revealed no effect of treatment (*F*(1, 16) = 1.312, *p* < 0.2689), but did reveal an effect of day (*F*(1,16) = 8.454, *p* < 0.0103) and a treatment x day interaction (*F*(1,16) = 6.582, *p* < 0.0207) on active lever presses. Holm-Bonferroni tests showed a significant increase in active lever presses on AD8 compared to AD1 in rats treated with vehicle (*p* < 0.0143), showing the expected incubation of cocaine seeking. Rats treated with RTIOX-276, however, did not show increases in cocaine seeking between AD8 and AD1 (*p* < 0.8232). We did not observe differences in lever pressing between vehicle and RTIOX-276 on AD1 (*p* < 0.8403) or AD8 (*p* < 0.1120). These results indicate that RTIOX-276 prevented incubation of cocaine seeking (Fig. 3G). Next, we examined if the number of lever presses on the AD1 predicted lever pressing on AD8 using a Pearson correlation and found no significant relationship between AD1 and AD8 lever pressing (*F*(1,16)  =  0.0967; R^2^  =  0.006, *p* < 0.7598; Fig. 3H). Together, these results suggest that acquisition, intake, and AD1 seeking behavior did not differ between incubated and non-incubated rats.

#### Hcrtr1 blockade reduced dopamine transmission and DAT biochemistry

**Fig. 4:**
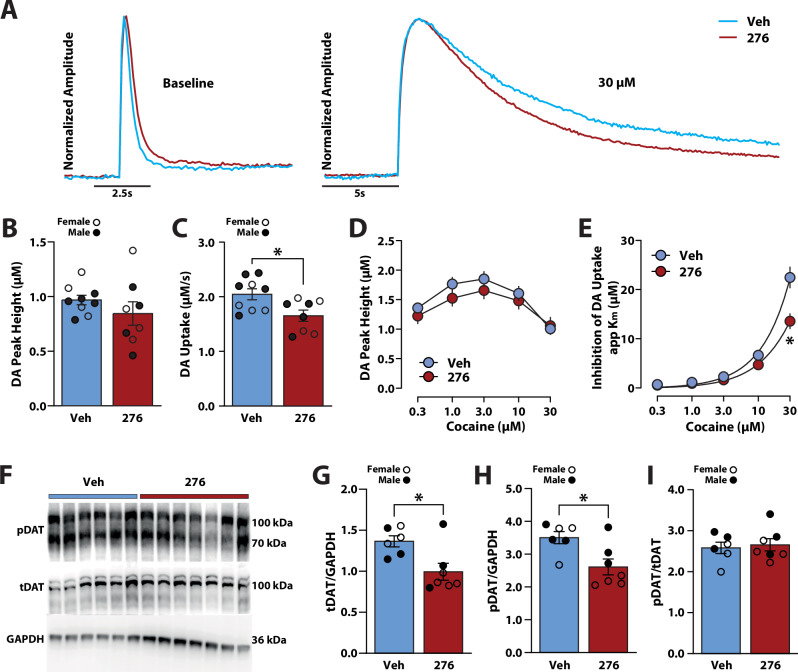
Hcrtr1 blockade decreased dopamine transmission and DAT expression. **A** Example dopamine traces for baseline measurements and following 30 μM cocaine. **B** Baseline dopamine peak height and **C** dopamine uptake, **D** cocaine-induced dopamine peak height and **E** inhibition of dopamine uptake (i.e, DAT sensitivity to cocaine; app Km) for rats treated with vehicle (Veh) or 20 mg/kg RTIOX-276 (276). **F** Example Western blots and **G** quantification of total membrane DAT (tDAT), **H** phosphorylated DAT (pDAT) over GAPDH, and **I** pDAT over tDAT (pDAT/tDAT) for rats treated with vehicle or RTIOX-276. Data shown as mean ± SEM. ○ females, ● males. Student’s t-tests *p < 0.05.

To determine if reductions in cocaine seeking following Hcrtr1 antagonism were associated with normalization of dopamine transmission, we measured dopamine peak height and uptake in the NAc 18 h following seeking tests on AD8 (Fig. 3A). A Student’s t-test revealed that Hcrtr1 antagonism did not significantly affect dopamine peak height (t(15) = 1.108, *p* < 0.2854) but did reduce dopamine uptake (t(15) = 2.703, *p* < 0.0164) compared to vehicle treatment (Fig. 4A–C).

To assess whether antagonism of Hcrtr1 reduced dopamine responses to cocaine, we conducted FSCV with bath application of cocaine. A two-way mixed design ANOVA with treatment (vehicle vs. RTIOX-276) as the between-subjects variable and cocaine concentration (0.3, 1, 3, 10, 30 μM) as the within-subjects variable showed a significant effect of concentration (Greenhouse-Geisser correction; *F*(1.910,28.65) = 58.35, *p* < 0.0001), but no significant effect of treatment (*F*(1,15) = 0.5757, *p* < 0.4598) or a treatment x concentration interaction (*F*(4,60) = 2.151, *p* < 0.0854) on cocaine-induced changes in dopamine peak height (Fig. 4D).

Next, we examined DAT sensitivity to cocaine using a two-way ANOVA with treatment (vehicle vs. RTIOX-276) as the between-subjects variable and cocaine concentration (0.3, 1, 3, 10, and 30 µM) as the within-subjects variable. This analysis revealed a significant effect of treatment (*F*(1,15) = 10.33, *p* < 0.0058), concentration (Greenhouse-Geisser correction; *F*(1.076,16.14) = 150.8, *p* < 0.0001), and a treatment x concentration interaction (*F*(1.076,16.14) = 9.825, *p* < 0.0056) on DAT sensitivity to cocaine (Fig. 4E). Holm-Bonferroni tests revealed that Hcrtr1 antagonism significantly reduced DAT sensitivity to cocaine at the 30 µM cocaine concentration (*p* < 0.0293), showed a trend for reduced effects at the 10 µM concentration (*p* < 0.0639), but did not affect DAT sensitivity at other concentrations (0.3 µM, *p* < 0.2966; 1 µM, *p* < 0.1953; 3 µM, *p* < 0.2350). Next, we examined if RTIOX-276 prevented changes in DAT expression observed following incubation of cocaine seeking. Student’s t-test showed that RTIOX-276 significantly reduced both tDAT (t(11) = 2.892, *p* < 0.0147), pDAT (t(11) = 2.841, *p* < 0.016), but not pDAT/tDAT (t(11) = 0.3579, *p* < 0.7272) expression (Fig. 4F–I). Lastly, we examined if lever pressing on AD8 was correlated with changes in dopamine transmission or DAT expression using Pearson correlations. We found no significant relationships between lever pressing and dopamine peak height (*F*(1,15)  =  0.5238; R^2^  =  0.0337, *p* < 0.4804), dopamine uptake (*F*(1,15)  =  2.140; R^2^  =  0.1248, *p* < 0.1642), dopamine peak height following 30 µM cocaine (*F*(1,15)  =  0.0170; R^2^  =  0.0011, *p* < 0.8980), or inhibition of dopamine uptake (*F*(1,15)  =  0.4345; R^2^  =  0.0282, *p* < 0.5198) following 30 µM cocaine (Supplemental Fig. 2A–D). Likewise, we did not observe significant correlations for tDAT (*F*(1,11)  =  0.1398; R^2^  =  0.0125, *p* < 0.7156), pDAT (*F*(1,11)  =  1.410; R^2^  =  0.1136, *p* < 0.2601), or pDAT/tDAT (*F*(1,11)  =  2.260; R^2^  =  0.1705, *p* < 0.1609) expression (Supplemental Fig. 2E–G).

## Discussion

We examined whether incubation of cocaine seeking is associated with changes in dopamine transmission and to what extent Hcrtr1 blockade early in abstinence reduces cocaine seeking and normalizes dopamine transmission later in abstinence. Results indicated that IntA to cocaine produced robust incubation in a subset of rats and that increases in dopamine uptake and dopamine responses to cocaine were observed preferentially in the group of rats that showed an incubation of cocaine seeking. Furthermore, we found that a single injection of the Hcrtr1 antagonist—RTIOX-276—on the first day of abstinence prevented incubation of cocaine seeking and aberrant dopamine adaptations one week later. Together, these findings suggest that Hcrtr1 antagonism early in abstinence exerts lasting therapeutic effects and thus may serve as a potential treatment strategy for reducing cocaine seeking and the likelihood of relapse.

### IntA followed by a brief abstinence period promoted incubation of cocaine seeking

Incubation of cocaine seeking, or the tendency of cue-induced cocaine seeking to increase throughout abstinence, has received significant attention as a key factor leading to relapse [5, 6, 8, 16, 17, 63–68]. Historically, most studies examining incubation of cocaine seeking have employed self-administration schedules that promote stable patterns of drug intake across sessions, such as the short access and long access schedules. While long access to cocaine reliably produces incubation of cocaine seeking [5, 69], evidence for incubation following short access schedules is limited, with only a few studies demonstrating time-dependent increases in cue-induced cocaine seeking under these conditions [66, 70]. By comparison, IntA to cocaine engenders temporally clustered, high intake rates that result in elevated peak cocaine concentrations [48]. This pattern of intake more closely models the binge-like, spiking cocaine intake patterns observed in humans [47, 48, 71] and may engender perceived drug scarcity, thereby enhancing its reward value [72]. Moreover, IntA has also been shown to produce enhanced motivation for cocaine, robust cue-induced reinstatement, and greater incubation of cocaine seeking despite lower total cocaine intake [47, 54, 73–75].

We previously compared the effects of IntA, long access and short access on incubation of cocaine seeking and found that after 28 days of abstinence, short access did not produce incubation. While both IntA and long access promoted incubation, IntA did so with comparatively lower cocaine intake, highlighting its efficiency in driving cocaine seeking [17]. Additionally, only IntA exposure increased dopamine uptake in the NAc compared to cocaine-naive controls, suggesting a unique neurochemical adaptation associated with intermittency of cocaine access. While these findings highlight the utility of IntA in driving incubation late in abstinence, cocaine-associated adaptations emerge soon after cocaine use cessation [76, 77] and may be predictive of relapse risk [78, 79]. Therefore, in the present studies we were interested in the degree to which IntA to cocaine elicited incubation of cocaine seeking early in abstinence, when the processes underlying incubation may be first emerging. We found that IntA to cocaine resulted in robust cocaine intake, but rats did not show escalation of intake over the 7 days of self-administration. While previous studies using IntA to cocaine have shown escalation of intake, most of these studies used a longer self-administration timeframe that may be conducive to escalation [54, 74, 80, 81]. Despite this absence of escalation, we nevertheless observed robust incubation of cocaine seeking 8 days into abstinence. Further, as discussed below, we also observed changes in dopamine transmission after IntA to cocaine, although this effect was observed preferentially in the subset of rats that increased cocaine seeking over the course of abstinence. When considered together, these findings suggest that incubation of cocaine seeking occurs independent of progressive increases in cocaine intake and that IntA reliably models individual differences in relapse vulnerability.

In the present studies, we used a within-subjects design in which rats were tested for cue-induced cocaine seeking on both AD1 and AD8. While this approach affords the opportunity to measure increases in cocaine seeking across abstinence, repeated testing also introduces potential limitations. For example, the initial test on AD1 may engage extinction learning in a manner that influences future behavior on AD8. Although our correlation analyses did not reveal a significant relationship between AD1 and AD8 responding, the possibility of experimental carryover effects cannot be excluded. This is particularly relevant since extinction can occur rapidly and may affect subsequent cue-induced responding. Notably, although most studies examining incubation of cocaine seeking have employed between-subjects designs, numerous studies have used a within-subjects design similar to what we employed in our studies [54–56, 70, 81–83]. Future studies incorporating both within- and between-subjects comparisons may be important for delineating the impact of test history on incubation.

### Changes in NAc dopamine transmission were tied to incubation of cocaine seeking

Incubation of cocaine seeking has been demonstrated to influence the activity of the mesolimbic dopamine pathway [12–15]. We and others have shown functional changes in dopamine transmission in the NAc during abstinence from cocaine [16, 17, 47, 73], including increases in dopamine uptake rate and DAT sensitivity to cocaine following 28 days of abstinence [17]. Consistent with these findings, here we observed increased dopamine uptake and DAT sensitivity to cocaine after 8 days of abstinence from IntA to cocaine. Despite these observations, it was not clear if these dopamine adaptations were a consequence of IntA cocaine exposure alone, abstinence from cocaine, or whether they were tied to incubation of cocaine seeking. To address this, we separated the IntA rats into those that developed incubated seeking and those that did not. Incubated rats (12 out of 18) and non-incubated rats (6 out of 18) did not differ in the amount of cocaine consumed or other measures of cocaine self-administration prior to abstinence, yet only the incubated rat group displayed increases in baseline dopamine uptake and exaggerated DAT sensitivity to cocaine. In fact, non-incubated rats demonstrated similar measures of dopamine transmission as cocaine-naive controls. While chronic cocaine use has been associated with persistent changes in mesolimbic dopamine function in both rodents and humans [47, 49, 84, 85], to our knowledge, this is the first study to demonstrate that dopamine disruptions are preferentially observed in subjects that expressed incubation of cocaine seeking.

As stated above, we previously demonstrated that after 28 days of abstinence both IntA and long access to cocaine engendered robust incubation of cocaine seeking while short access did not. In those studies, we also found that short access had no effect on dopamine transmission, but IntA and long access produced unique dopamine transmission changes [17]. For example, long access increased DAT sensitivity to cocaine without affecting baseline uptake, while IntA induced a broader profile of changes by enhancing both baseline dopamine uptake and DAT sensitivity to cocaine. These findings highlight that intermittency of cocaine exposure may produce more robust alterations in dopamine transmission than either short access or long access to cocaine. Consistent with these observations, in the present studies we found that IntA to cocaine promoted incubation and associated changes in dopamine transmission 8 days into abstinence and that these dopamine effects were observed preferentially in rats that individually increased cocaine seeking. While we did not directly compare incubation and dopamine changes across schedules of reinforcement herein, prior work suggests that both long access and short access to cocaine can produce moderate increases in drug seeking during the first 15 days of abstinence [5, 50, 52, 55, 56, 67, 70, 86, 87]. The extent to which these behavioral changes are tied to alterations in dopamine transmission, however, remains largely unknown. Collectively, these findings indicate that IntA to cocaine models early abstinence-related dopamine and behavioral adaptations linked to relapse vulnerability and therefore may provide a useful translational platform for testing interventions to prevent incubation of cocaine seeking.

### Hcrtr1 blockade prevented incubation of cocaine seeking and normalized dopamine transmission in the NAc

A principal challenge in treating cocaine use disorder is reducing intensification of cocaine craving during periods of abstinence [5, 67]. Given our observation that incubation of cocaine seeking was accompanied by dopamine adaptations, we hypothesized that normalizing dopamine adaptations would prevent incubation of cocaine seeking. Extensive evidence indicates that the hypocretin system influences cocaine-associated behavior and that these effects involve actions on dopamine transmission, including changes in dopamine uptake, DAT phosphorylation, and dopamine responses to cocaine [23, 24, 26, 27, 32–34, 39, 42, 88–90]. Despite this evidence, the therapeutic potential of Hcrtr1 blockade for treating incubation of cocaine seeking has not been previously reported. In the second set of studies, we administered the Hcrtr1 antagonist—RTIOX-276—on the first day of abstinence. This timepoint was chosen to maximize potential therapeutic benefit by curtailing the development of incubation of cocaine seeking, rather than reversing its expression. Additionally, because adherence to treatments for substance use disorder tends to wane over the course of abstinence, an effective treatment for reducing craving early in abstinence would be highly valuable for decreasing rates of relapse [91, 92]. We found that administration of RTIOX-276 immediately following seeking tests on AD1 significantly decreased cue-induced cocaine seeking one week later on AD8. Moreover, when examining if these behavioral effects were linked to changes in dopamine transmission, we observed that RTIOX-276 reduced the expected enhancement in dopamine transmission observed following IntA to cocaine. Notably, in the vehicle-treated group, only one rat failed to incubate, which is a considerably smaller proportion compared to the first set of studies, where 6 out of 18 rats did not incubate. This marked difference in proportions suggests potential cohort-specific variations that may limit generalizability across these studies. Such variability underscores the need for additional work to establish the mechanistic basis of incubation of cocaine seeking across experimental contexts.

The circuits through which IntA to cocaine influences dopamine transmission have not been fully explored. However, accumulating evidence suggests that extended abstinence from IntA to drugs of abuse increases c-fos expression in hypocretin neurons [80] and/or increases hypocretin neuron number [80, 93], both of which may reflect hypocretin system plasticity that results in enhanced peptide production from ‘reserve’ neurons. Given that delivery of Hcrt-1 peptide into the VTA enhances dopamine neuron activity, DAT sensitivity to cocaine, and cocaine associated behavior [19, 39], IntA-induced elevations in hypocretin activity would be expected to increase VTA dopamine neuron activity and enhance behavioral responses to cocaine. Importantly, pharmacological blockade of Hcrtr1 with RTIOX-276 should attenuate these IntA-induced interactions by reducing the effects of elevated hypocretin activity in the VTA, thereby normalizing dopamine transmission and reducing cocaine seeking.

The finding that a single Hcrtr1 antagonist treatment has prolonged behavioral and neurochemical effects is intriguing, although the mechanisms of action are not clear. While most prior studies on hypocretin regulation of cocaine-associated behavior have been restricted to the acute, onboard pharmacological effects of Hcrtr1 antagonists, a limited set of studies have reported lasting effects of Hcrtr1 antagonists in the context of drug abuse [42, 94–96]. For instance, evidence from our laboratory indicates that Hcrtr1 antagonism reduced cocaine self-administration and attenuated DAT sensitivity to cocaine for up to 24 h after treatment, an effect that is beyond the pharmacological availability of the Hcrtr1 antagonist [59]. Further, we recently reported that a single treatment with RTIOX-276 on the first day of abstinence reduced demand for cocaine after 7 days of abstinence from IntA to cocaine [43]. The mechanisms underlying lasting changes in behavior and dopamine transmission following Hcrtr1 antagonists remain unclear. However, it is possible that Hcrtr1 antagonism exerts acute reductions in dopamine neuron activity within the VTA that lead to lasting effects. Indeed, previous studies have shown that cocaine exposure induces changes in glutamate receptors on VTA neurons within hours of exposure [97, 98] and significantly increases dopamine shortly after cocaine intake ceases [99]. Further, there is considerable evidence that Hcrtr1 antagonism reduces dopamine neuron activity [22, 25, 35–41] and dopamine transmission in the NAc [23, 24, 28, 42]. In this capacity, Hcrtr1 antagonism may prevent or reverse the effects of cocaine exposure on VTA dopamine neuron activity and downstream NAc dopamine signaling during a sensitive period early in abstinence, thus preventing the behavioral and biochemical changes observed with IntA to cocaine.

In this context, the timing of RTIOX-276 administration immediately after the AD1 seeking test is noteworthy, as it raises the possibility that Hcrtr1 antagonism may exert its behavioral effects by disrupting the consolidation of extinction-related memories. Given that Hcrtr1 blockade produces lasting changes in dopamine transmission [42, 43], it is possible that memory consolidation in the context of cocaine seeking may be dopamine dependent. Future studies will be required to establish if there is a critical window for disrupting dopamine transmission that impacts later cocaine-seeking behavior.

### Alterations in DAT expression and phosphorylation may underlie changes in dopamine transmission

Accumulating evidence suggests that phosphorylation of DAT at threonine 53 is associated with faster dopamine uptake, increased DAT sensitivity to cocaine, and increased motivation for cocaine [42, 100–103]. In the current studies we did not observe differences in the proportion of total membrane DATs that were phosphorylated at the threonine 53 site. Nonetheless, absolute increases in pDAT levels were preferentially observed in rats that displayed incubation of cocaine seeking, likely due to increases in tDAT levels. These effects matched our FSCV findings in which only rats that displayed incubation of cocaine seeking displayed increases in dopamine uptake and DAT sensitivity to cocaine. The effects of Hcrtr1 blockade on dopamine transmission are also consistent with changes in DAT biochemistry. Even without a change in pDAT/tDAT, RTIOX-276 reduced both pDAT and total membrane DAT expression after abstinence from IntA to cocaine. When considered together, these findings suggest that changes in dopamine uptake and DAT sensitivity to cocaine observed in rats with incubated seeking are likely mediated by increased levels of DAT and DAT phosphorylation, similar to what we have suggested previously [42, 58, 59]. The observed results also support the premise that RTIOX-276 reduces cocaine seeking by preventing these dopamine adaptations. In addition to DAT phosphorylation state or total membrane expression, other mechanisms may contribute to alterations in DAT function [104]. For example, several reports suggest that shifts in the proportion of inward/outward facing DATs [105], changes in oligomer/monomer ratios [86, 87], dimerization with sigma receptor [106], or phosphorylation of DAT serine sites [107, 108] impact the efficiency of dopamine uptake. Therefore, although our findings demonstrate a link between DAT function, DAT expression, and DAT phosphorylation at threonine 53, other mechanisms are likely to be involved [104].

## Summary

We demonstrated that IntA to cocaine engenders dopamine adaptations that underlie incubation of cocaine seeking and that Hcrtr1 antagonism early in abstinence prevents these dopamine adaptations and exerts lasting reductions in cue-induced cocaine seeking. Given that early abstinence represents a particularly vulnerable period for treating cocaine use disorder, these findings suggest that Hcrtr1 antagonism early in abstinence – when cocaine craving begins to develop – may serve as a potentially valuable therapeutic option for decreasing craving and reducing the propensity for relapse.

## Supplementary information

Supplemental Tables and Figures

## Data Availability

Data will be made available upon reasonable request.
