# Supplementary material for: Hypocretin receptor 1 blockade early in abstinence prevents incubation of cocaine seeking and normalizes dopamine transmission
Source: Neuropsychopharmacology. 2026 Feb 4;51(6):1123–34. doi: 10.1038/s41386-025-02315-9 (PMC13125265; doi:10.1038/s41386-025-02315-9)
Supplement: Supplementary file 1 — Supplemental Tables and Figures [file 41386_2025_2315_MOESM1_ESM.pdf]

Table I

**Measure**	**Dependent Variable**	**Test**	**Statistic**
Days to Acquire	Sex	Unpaired Student's t-test	t(16) = 0.9928, $p<0.3356$
Active Lever Pressing	Sex<br>Day<br>Day x Sex	Two-way mixed design ANOVA	$F(1, 16) = 0.1855$, $p<0.6724$<br>$F(3.538, 56.61) = 1.420$, $p<0.2427$<br>$F(3.538, 56.61) = 0.3519$, $p<0.8194$
IntA Escalation of Intake (mg/kg)	Sex<br>Day<br>Day x Sex	Two-way mixed design ANOVA	$F(1, 16) = 2.105$, $p<0.1661$<br>$F(1, 16) = 0.3449$, $p<0.5652$<br>$F(1, 16) = 0.6355$, $p<0.8012$
LgA Intake (mg/kg)	Sex	Unpaired Student's t-test	t(16) = 1.098, $p<0.2887$
IntA Intake (mg/kg)	Sex	Unpaired Student's t-test	t(16) = 1.345, $p<0.1975$
Total Intake (mg/kg)	Sex	Unpaired Student's t-test	t(16) = 1.322, $p<0.2047$
Cue-Induced Seeking	Sex<br>Abstinence Day<br>Sex x Abstinence Day	Two-way mixed design ANOVA	$F(1, 16) = 1.408$, $p<0.2528$<br>$F(1, 16) = 7.920$, $p<0.0125$*<br>$F(1, 16) = 0.6645$, $p<0.4269$
Proportion of Females and Males that Incubated	(blank) / Inc / Non-Inc<br>Female / 4 / 2<br>Male / 7 / 4	Contingency Chi-Square Test for Independence	($\chi^2(1, n = 17) = 0.01561$, $p<0.9006$)
Baseline Dopamine Release	Sex<br>Condition<br>Sex x Condition	Two-way mixed design ANOVA	$F(1, 27) = 0.1308$, $p<0.7204$<br>$F(1, 27) = 5.511$, $p<0.0265$*<br>$F(1, 27) = 1.039$, $p<0.3172$
Baseline Dopamine Uptake	Sex<br>Condition<br>Sex x Condition	Two-way mixed design ANOVA	$F(1, 27) = 0.0652$, $p<0.8004$<br>$F(1, 27) = 8.626$, $p<0.0067$*<br>$F(1, 27) = 1.709$, $p<0.2022$
Cocaine Effect on Dopamine Release	Sex<br>Concentration<br>Condition<br>Sex x Concentration<br>Sex x Condition<br>Concentration x Condition<br>Sex x Concentration x Condition	Three-way mixed design ANOVA	$F(1, 26) = 1.063$, $p<0.312$<br>$F(2.085, 54.20) = 94.15$, $p<0.0001$***<br>$F(1, 26) = 0.0084$, $p<0.9277$<br>$F(2.085, 54.2) = 0.3279$, $p<0.7307$<br>$F(1, 26) = 2.424$, $p<0.1315$<br>$F(2.085, 54.2) = 4.117$, $p<0.0203$*<br>(2.085, 54.2) = 1.156, $p<0.3238$
Cocaine Effect on Dopamine Uptake	Sex<br>Concentration<br>Condition<br>Sex x Concentration<br>Sex x Condition<br>Concentration x Condition<br>Sex x Concentration x Condition	Three-way mixed design ANOVA	$F(1, 26) = 0.1458$, $p<0.7057$<br>$F(1.112, 28.90) = 299.4$, $p<0.0001$***<br>$F(1, 26) = 9.392$, $p<0.005$**<br>$F(1.112, 28.90) = 0.3863$, $p<0.5611$<br>$F(1, 26) = 0.3278$, $p<0.5719$<br>$F(1.112, 28.90) = 8.598$, $p<0.0053$**<br>$F(1.112, 28.90) = 0.2617$, $p<0.6378$
tDAT/GAPDH	Sex<br>Condition<br>Sex x Condition	Two-way mixed design ANOVA	$F(1, 14) = 0.7715$, $p<0.3946$<br>$F(1, 14) = 1.639$, $p<0.2213$<br>$F(1, 14) = 0.00001$, $p<0.9976$
pDAT/GAPDH	Sex<br>Condition<br>Sex x Condition	Two-way mixed design ANOVA	$F(1, 14) = 1.176$, $p<0.2964$<br>$F(1, 14) = 1.004$, $p<0.3334$<br>$F(1, 14) = 0.0064$, $p<0.9373$
pDAT/tDAT	Sex<br>Condition<br>Sex x Condition	Two-way mixed design ANOVA	$F(1, 14) = 0.109$, $p<0.7474$<br>$F(1, 14) = 0.0848$, $p<0.7752$<br>$F(1, 14) = 0.0011$, $p<0.9735$

**Table II**			
**Measure**	**Dependent Variable**	**Test**	**Statistic**
Days to Acquire	Sex<br>Treatment<br>Sex x Treatment	Two-way mixed design ANOVA	$F(1, 14) = 0.2026, p<0.6595$<br>$F(1, 14) = 0.3026, p<0.5909$<br>$F(1, 14) = 0.9028, p<0.3581$
Active Lever Pressing	Sex<br>Days<br>Treatment<br>Sex x Days<br>Sex x Treatment<br>Days x Treatment<br>Sex x Days x Treatment	Three-way mixed design ANOVA	$F(1, 14) = 0.2942, p<0.5961$<br>$F(3.476, 48.66) = 1.564, p<0.2048$<br>$F(1, 14) = 0.9238, p<0.3528$<br>$F(3.476, 48.66) = 0.4748, p<0.7284$<br>$F(1, 14) = 1.138, p<0.3041$<br>$F(3.476, 48.66) = 1.552, p<0.2078$<br>$F(3.476, 48.66) = 0.8245, p<0.5018$
IntA Escalation of Intake (mg/kg)	Sex<br>Days<br>Treatment<br>Sex x Days<br>Sex x Treatment<br>Days x Treatment<br>Sex x Days x Treatment	Three-way mixed design ANOVA	$F(1, 14) = 0.8256, p<0.3789$<br>$F(1, 14) = 3.612, p<0.0782$<br>$F(1, 14) = 1.403, p<0.2560$<br>$F(1, 14) = 0.1348, p<0.7190$<br>$F(1, 14) = 1.213, p<0.2893$<br>$F(1, 14) = 0.1866, p<0.6724$<br>$F(1, 14) = 1.971, p<0.1822$
LgA Intake (mg/kg)	Sex<br>Treatment<br>Sex x Treatment	Two-way mixed design ANOVA	$F(1, 14) = 0.0057, p<0.9407$<br>$F(1, 14) = 0.7522, p<0.4004$<br>$F(1, 14) = 0.0023, p<0.9622$
IntA Intake (mg/kg)	Sex<br>Treatment<br>Sex x Treatment	Two-way mixed design ANOVA	$F(1, 14) = 0.3170, p<0.5823$<br>$F(1, 14) = 1.243, p<0.2836$<br>$F(1, 14) = 0.9985, p<0.3346$
Total Intake (mg/kg)	Sex<br>Treatment<br>Sex x Treatment	Two-way mixed design ANOVA	$F(1, 14) = 0.4941, p<0.4936$<br>$F(1, 14) = 1.624, p<0.2233$<br>$F(1, 14) = 1.107, p<0.3105$
Cue-Induced Seeking	Sex<br>Abstinence Day<br>Treatment<br>Sex x Abstinence Day<br>Sex x Treatment<br>Abstinence Day x Treatment<br>Sex x Abstinence Day x Treatment	Two-way mixed design ANOVA	$F(1, 14) = 0.1874, p<0.6717$<br>$F(1, 14) = 8.223, p<0.0124$*<br>$F(1, 14) = 1.171, p<0.2974$<br>$F(1, 14) = 3.711, p<0.0746$<br>$F(1, 14) = 0.008134, p<0.9294$<br>$F(1, 14) = 6.978, p<0.0193$*<br>$F(1, 14) = 0.4851, p<0.4975$
Proportion of Females and Males that Incubated	(blank) / Inc / Non-Inc<br>Female / 5 / 3<br>Male / 8 / 2	Chi-Square Test for Independence	($\chi^2(1, n = 18) = 0.6785, p<0.4101$)
Baseline Dopamine Release	Sex<br>Treatment<br>Sex x Treatment	Two-way mixed design ANOVA	$F(1, 13) = 0.6731, p<0.4268$<br>$F(1, 13) = 1.184, p<0.2963$<br>$F(1, 13) = 0.0579, p<0.8135$
Baseline Dopamine Uptake	Sex<br>Treatment<br>Sex x Treatment	Two-way mixed design ANOVA	$F(1, 13) = 0.9803, p<0.3402$<br>$F(1, 13) = 6.662, p<0.0228$*<br>$F(1, 13) = 0.8328, p<0.3781$
Cocaine Effect on Dopamine Release	Sex<br>Concentration<br>Treatment<br>Sex x Concentration<br>Sex x Treatment<br>Concentration x Treatment<br>Sex x Concentration x Treatment	Three-way mixed design ANOVA	$F(1, 13) = 3.123, p<0.1007$<br>$F(1.944, 25.28) = 56.08, p<0.0001$***<br>$F(1, 13) = 0.8358, p<0.3772$<br>$F(4, 52) = 0.2232, p<0.9243$<br>$F(1, 13) = 0.1979, p<0.6638$<br>$F(4, 52) = 2.017, p<0.1057$<br>$F(4, 52) = 1.332, p<0.2706$
Cocaine Effect on Dopamine Uptake	Sex<br>Concentration<br>Treatment<br>Sex x Concentration<br>Sex x Treatment<br>Concentration x Treatment<br>Sex x Concentration x Treatment	Three-way mixed design ANOVA	$F(1, 13) = 0.2455, p<0.6285$<br>$F(1.088, 14.14) = 152.5, p<0.0001$***<br>$F(1, 13) = 9.714, p<0.0082$**<br>$F(4, 52) = 0.5732, p<0.6832$<br>$F(1, 13) = 1.816, p<0.2008$<br>$F(4, 52) = 9.396, p<0.0001$***<br>$F(4, 52) = 1.856, p<0.1323$
tDAT/GAPDH	Sex<br>Treatment<br>Sex x Treatment	Two-way mixed design ANOVA	$F(1, 9) = 0.2718, p<0.6147$<br>$F(1, 9) = 8.235, p<0.0185$*<br>$F(1, 9) = 2.751, p<0.1315$
pDAT/GAPDH	Sex<br>Treatment<br>Sex x Treatment	Two-way mixed design ANOVA	$F(1, 9) = 6.133, p<0.0352$*<br>$F(1, 9) = 11.57, p<0.0078$**<br>$F(1, 9) = 3.125, p<0.1109$
pDAT/tDAT	Sex<br>Treatment<br>Sex x Treatment	Two-way mixed design ANOVA	$F(1, 9) = 5.252, p<0.0476$*<br>$F(1, 9) = 0.3237, p<0.5833$<br>$F(1, 9) = 0.00004, p<0.9947$

Discussion

Sex differences have been observed across various aspects of cocaine self-administration, suggesting that females acquire self-administration behavior more quickly (Lynch and Carroll, 1999; Becker and Koob, 2016), are more motivated to self-administer cocaine (Roberts et al., 1987; Roberts et al., 1989; Carroll et al., 2002; Lynch, 2006; Black et al., 2023), and engage in higher cocaine seeking after intermittent access to cocaine (Nicolas et al., 2021). Despite these observations, and similar to our previous publication on incubation of cocaine seeking (Alonso et al., 2022), we observed no significant sex differences in acquisition, cocaine intake, or cocaine seeking—although there was a trend for sex differences in acquisition and seeking for the Hcrtr1 studies prior to RTIOX-276 treatment. Previous studies have also observed sex differences in baseline and cocaine-induced dopamine transmission, with females generally demonstrating faster dopamine uptake rates (Walker et al., 2000; Calipari et al., 2017; Black et al., 2023). Here we found no differences in fast scan cyclic voltammetry measures of dopamine transmission. However, we did observe a sex difference in pDAT expression in the Hcrtr1 studies suggesting that males may have slightly higher pDAT levels than females. Importantly, across all studies there were no significant interactions between sex and any behavioral, voltammetry, or biochemistry measure, indicating that rats of both sexes responded similarly to cocaine exposure and Hcrtr1 blockade.

References Cited

Alonso IP, O'Connor BM, Bryant KG, Mandalaywala RK, España RA (2022) Incubation of cocaine craving coincides with changes in dopamine terminal neurotransmission. Addiction Neuroscience:100029.

Becker JB, Koob GF (2016) Sex Differences in Animal Models: Focus on Addiction. Pharmacol Rev 68:242-263.

Black EM, Samels SB, Xu W, Barson JR, Bass CE, Kortagere S, España RA (2023) Hypocretin / Orexin Receptor 1 Knockdown in GABA or Dopamine Neurons in the Ventral Tegmental Area Differentially Impact Mesolimbic Dopamine and Motivation for Cocaine. Addict Neurosci 7.

Calipari ES, Juarez B, Morel C, Walker DM, Cahill ME, Ribeiro E, Roman-Ortiz C, Ramakrishnan C, Deisseroth K, Han MH, Nestler EJ (2017) Dopaminergic dynamics underlying sex-specific cocaine reward. Nat Commun 8:13877.

Carroll ME, Morgan AD, Lynch WJ, Campbell UC, Dess NK (2002) Intravenous cocaine and heroin self-administration in rats selectively bred for differential saccharin intake: phenotype and sex differences. Psychopharmacology (Berl) 161:304-313.

Lynch WJ (2006) Sex differences in vulnerability to drug self-administration. Exp Clin Psychopharmacol 14:34-41.

Lynch WJ, Carroll ME (1999) Sex differences in the acquisition of intravenously self-administered cocaine and heroin in rats. Psychopharmacology (Berl) 144:77-82.

Nicolas C, Russell TI, Shaham Y, Ikemoto S (2021) Dissociation Between Incubation of Cocaine Craving and Anxiety-Related Behaviors After Continuous and Intermittent Access Self-Administration. Front Neurosci 15:824741.

Roberts DC, Dalton JC, Vickers GJ (1987) Increased self-administration of cocaine following haloperidol: effect of ovariectomy, estrogen replacement, and estrous cycle. Pharmacol Biochem Behav 26:37-43.

Roberts DC, Bennett SA, Vickers GJ (1989) The estrous cycle affects cocaine self-administration on a progressive ratio schedule in rats. Psychopharmacology (Berl) 98:408-411.

Walker QD, Rooney MB, Wightman RM, Kuhn CM (2000) Dopamine release and uptake are greater in female than male rat striatum as measured by fast cyclic voltammetry. Neuroscience 95:1061-1070.

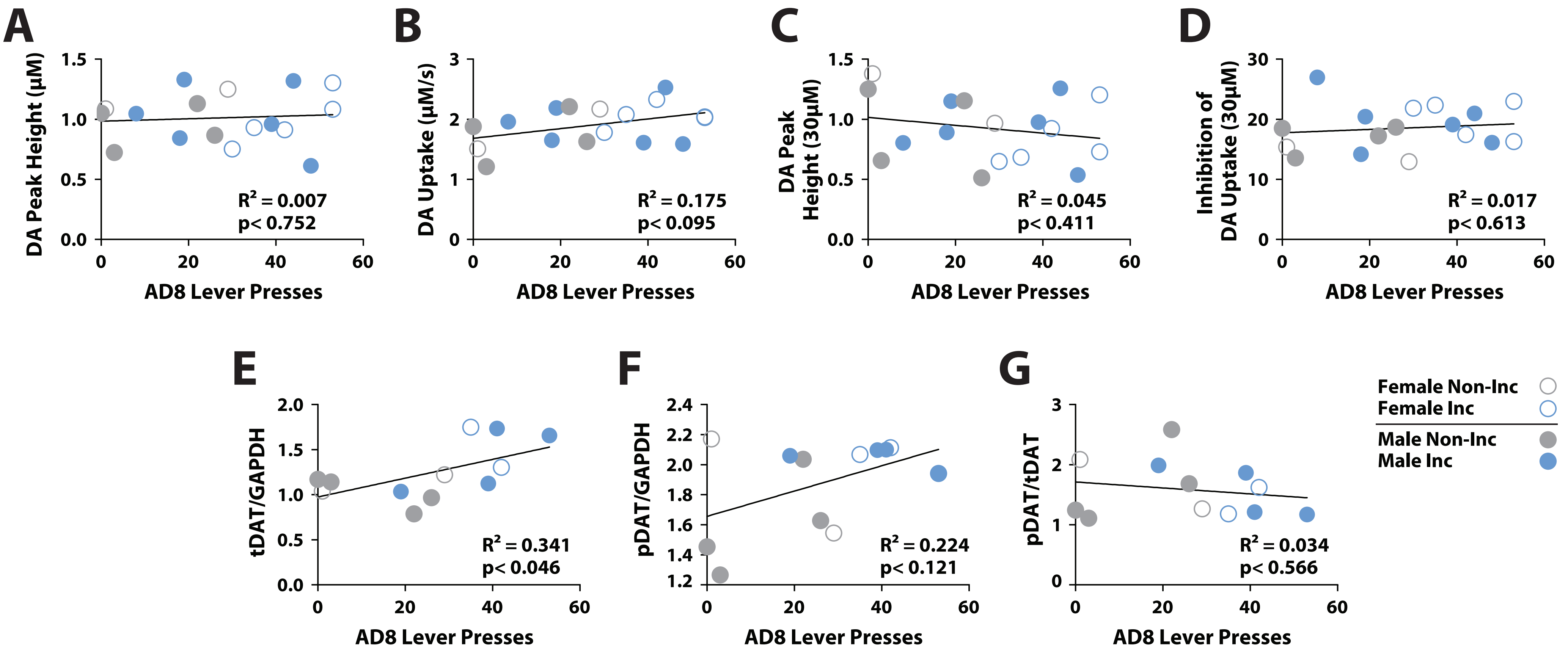

Supplemental Figure 1. Pearson correlations between lever pressing on abstinence day 8 and dopamine measures. (A) Baseline dopamine peak height, (B) baseline dopamine uptake, (C) cocaine-induced dopamine peak height (at 30μM), (D) cocaine-induced inhibition of dopamine uptake (at 30μM), (E) total membrane DAT over GAPDH (tDAT/GAPDH), (F) phosphorylated DAT over GAPDH (pDAT/GAPDH), and (G) pDAT over tDAT (pDAT/tDAT) for naive, non-incubated (Non-Inc), and incubated (Inc) rats. Data shown as mean±SEM.

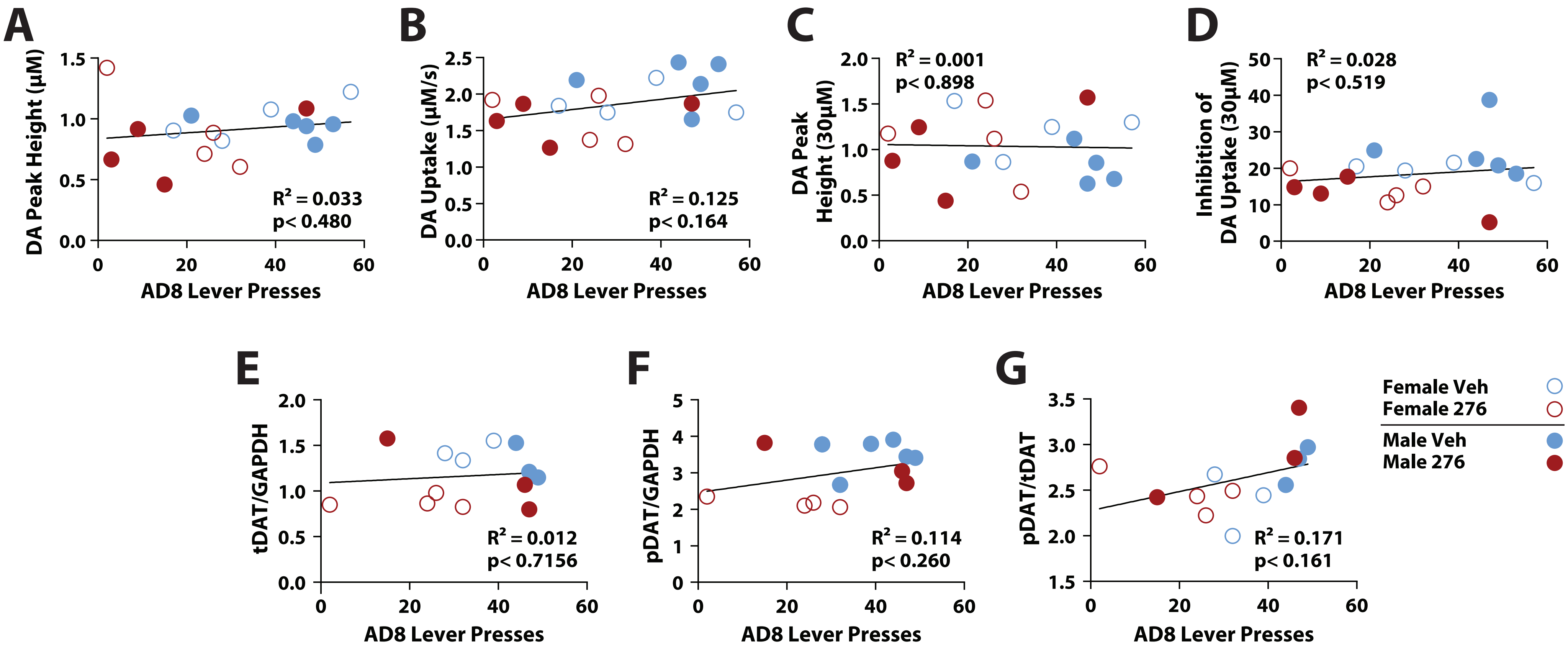

Supplemental Figure 2. Pearson correlations between lever pressing on abstinence day 8 and dopamine measures. (A) Baseline dopamine peak height, (B) baseline dopamine uptake, (C) cocaine-induced dopamine peak height (at 30µM), (D) cocaine-induced inhibition of dopamine uptake (at 30µM), (E) total membrane DAT over GAPDH (tDAT/GAPDH), (F) phosphorylated DAT over GAPDH (pDAT/GAPDH), and (G) pDAT over tDAT (pDAT/tDAT) for rats treated with vehicle and RTIOX-276. Data shown as mean±SEM.
